# Efficacy and Safety of Nintedanib in Patients with Connective Tissue Disease-Interstitial Lung Disease (CTD-ILD): A Real-World Single Center Experience

**DOI:** 10.3390/diagnostics13071221

**Published:** 2023-03-23

**Authors:** Maria Boutel, Afroditi Boutou, Georgia Pitsiou, Alexandros Garyfallos, Theodoros Dimitroulas

**Affiliations:** 1 Fourth Department of Internal Medicine, Hippokration University Hospital, Medical School, Aristotle University of Thessaloniki, 54642 Thessaloniki, Greece; mmpoutelag@gmail.com (M.B.); garyalex@auth.gr (A.G.); 2 Department of Respiratory Medicine, G. Papanikolaou Hospital, Aristotle University of Thessaloniki, 57010 Pylaia-Chortiatis, Greece; afboutou@yahoo.com; 3 Department of Respiratory Failure, G. Papanikolaou Hospital, Aristotle University of Thessaloniki, 57010 Pylaia-Chortiatis, Greece; gpitsiou@auth.gr

**Keywords:** connective tissue interstitial lung disease, CTD-ILD, ILD, nintedanib, real-world data

## Abstract

Connective Tissue Disease-Interstitial Lung Disease (CTD-ILD) is a severe and fatal manifestation of systemic autoimmune disorders. Therapies rely on immunomodulators but their efficacy in ILD progression remains uncertain. Nintedanib, an antifibrotic agent that slows pulmonary function decline, has been approved for CTD-ILD treatment. The aim of this study was to assess the effectiveness and safety of nintedanib in CTD-ILD patients in a real-world data setting. A single-center, retrospective, and descriptive analysis of CTD-ILD patients treated with nintedanib from June 2019 to November 2022 was performed. The assessment of nintedanib treatment’s efficacy was judged solely on the evolution of pulmonary function tests (PFTs), which were evaluated before and after treatment. Twenty-one patients (67% females, median age 64 years (IQR = 9) with CTD-ILD (systemic sclerosis *n* = 9, rheumatoid arthritis *n* = 5, dermatomyositis *n* = 4, juvenile rheumatoid arthritis *n* = 1, undifferentiated CTD *n* = 1, interstitial pneumonia with autoimmune features *n* = 1), 18 of whom were on concomitant immunosuppressives, had a median follow-up period of 10 months (IQR = 5). PFTs before and after treatment did not significantly differ. The mean FVC% difference was +0.9 (sd = 7.6) and the mean DLco% difference was +3.4 (sd = 12.6), suggesting numerical improvement of PFTs. The average percentage change was −0.3% and +7.6% for FVC% and DLco%, respectively, indicating stabilization of lung function. Our real-world data across a broad spectrum of CTD-ILD suggest that nintedanib could be beneficial in combination with immunosuppressives in slowing the rate of lung function decline.

## 1. Introduction

Interstitial Lung Diseases (ILDs) are a large group of several relatively different pulmonary pathologies. Many ILDs are characterized by fibrosis and lung parenchyma damage, especially of the interstitium, as well as various degrees of inflammation [1]. The fibrosis that appears in many ILDs is a result of the scarring of the lung tissue that provokes abnormal healing mechanisms and excessive matrix accumulation [2,3]. Connective Tissue Disease-ILD (CTD-ILD) is a severe pulmonary manifestation of connective tissue diseases (CTDs) [4]. Despite the different clinical entities, such as systemic sclerosis (SSc), rheumatoid arthritis (RA), mixed CTD, and others, CTD-ILDs share similarities in the pathophysiology, clinical symptoms, and outcomes including progressive lung failure, dyspnea, and poor prognosis [5,6].

The most prevalent connective tissue diseases that develop CTD-ILD are rheumatoid arthritis (RA) and systemic sclerosis (SSc). RA conveys high rates of inflammation and affects several organs. RA-ILD affects almost 7.7% of RA patients, 25% of whom present with serious pulmonary decline already from diagnosis, whereas 25% of RA individuals will develop severe lung impairment in the first 5 years after diagnosis [7]. With a mean survival range of 5–8 years, ILD is a severe pulmonary complication of RA that accounts for 10–20% of disease-related mortality [8]. Major risk factors for ILD developing in RA are usually male sex, late onset of disease (usually people older than 60 years), seropositivity—especially the presence of high levels of anti-citrullinated protein antibodies (anti-CCP)—and active RA disease [7,9]. SSc is characterized by skin and internal organs fibrosis as well as vasculopathy and immune activation. The majority of SSc patients develop ILD during the course of the disease [10]. ILD represents one of the leading causes of all SSc-related deaths, with age, diffuse subtype, and the presence of a positive Scl-70 antibody having been recognized as risk factors [11]

Lung involvement is a significant prognostic factor of CTD as it is associated with an unfavorable outcome, thus great emphasis on an early diagnosis is given. The diagnosis of CTD-ILD is based on imaging investigations, lung function tests, and patient-reported dyspnea. The imaging of CTD-ILD is mainly conducted by high-resolution computed tomographic scan (HRCT) that focuses on usual interstitial pneumonia (UIP) and fibrosing nonspecific interstitial pneumonia (fNSIP) patterns [12]. The UIP pattern is more common in RA ILD and is linked with a worse prognosis, while the fNSIP pattern could be seen in every CTD-ILD. Lately, ultrasound is used for CTD-ILD screening too [13]. Lung function tests predominantly assess the decrease in forced vital capacity (FVC) and carbon monoxide diffusing capacity (DLco). Specifically, a ≥10% relative decline in FVC or ≥5% to <10% relative decline in FVC and ≥15% relative decline in DLco suggest disease progression and constitutes an indication of treatment initiation or alteration [14]. Additionally, patients’ subjective feelings of dyspnea and cough occurrence are important assessments conducted either via health and quality of life questionnaires, such as the Saint George’s Respiratory Questionnaire (SGRQ) and the Health Assessment Questionnaire (HAQ)-DI (disability index), or as patient-reported outcomes (PRO) [15]. The aforementioned diagnostic tools are also used to monitor and follow up on patients with ILD.

Due to their common clinical phenotype and refractory nature, CTD-ILDs are generally grouped together, mainly in view of common treatment strategies including steroids, immunosuppressive and antifibrotic agents [16,17,18]. Currently, there is no certain treatment protocol and thus management of CTD-ILD varies from frequent surveillance to the administration of steroid and immunomodulatory drugs. Therapeutic choices are based on the underlying CTD, the extent and severity of fibrosis, and the degree of reported dyspnea [19]. In detail, the most frequent immunosuppressive therapeutic options are prednisolone, cyclophosphamide (CYC), and mycophenolate mofetil (MMF), as well as biologic regimens such as tocilizumab, rituximab, and abatacept. CYC was the first official therapy for SSc-ILD and later MMF was proven to be equally effective and less toxic [20]. Tocilizumab has been recently viewed as a treatment choice, especially in the early, preclinical disease stages of SSc-ILD with an inflammatory component defined by high C-reactive protein (CRP) [21]. Rituximab is a new treatment option for stabilizing and improving CTD-ILD demonstrated in the recently published RECITAL study. Rituximab presented fewer side effects compared to CYC and patients reported improvement in quality of life, establishing it as a beneficial and well-tolerated treatment choice [22,23]. Another recent treatment option for RA-related ILD is abatacept. Relevant studies have concluded that abatacept has the potential to stabilize ILD with a lower risk of infection compared to other biologic disease-modifying regimens in RA patients [24]. Autologous hematopoietic stem cell transplantation and lung transplantation are also emerging options for treatments of SSc-ILD in refractory cases [25]. However, despite the increasing armamentarium of immunosuppressive drugs, CTD-ILD outcomes remain poor, and antifibrotic agents such as pirfenidone and nintedanib have been investigated as an additional therapeutic approach to this condition [26].

Nintedanib is a potent oral tyrosine kinase inhibitor that suppresses different processes of lung fibrosis [27]. The TOMORROW trial—a Phase II randomized, placebo-controlled trial—and the INPULSIS trials—two duplicate, phase III trials—established nintedanib as an antifibrotic treatment for idiopathic pulmonary fibrosis, as patients who received nintedanib had a significant improvement in FVC decline compared to the placebo group [28,29]. Recent high-quality randomized clinical trials have demonstrated the efficacy of nintedanib in reducing the progression of pulmonary fibrosis and the deterioration of pulmonary function tests (PFTs) in CTD-ILD [17,30,31]. Nintedanib has been approved for the treatment of CTD-ILD in combination with immunosuppressives, whilst monotherapy with antifibrotic regimens is not currently recommended. Despite promising data from the randomized controlled trials mentioned above, the outcomes of treatment with nintedanib in routine clinical practice remain unknown.

The aim of the current study was to evaluate, via the evolution of lung function parameters, the efficacy and safety profile of nintedanib in CTD-ILD patients with progressive fibrotic phenotype requiring treatment with antifibrotic agents in a real-world data setting.

## 2. Materials and Methods

### 2.1. Patients

We retrospectively reviewed the medical files of all CTD-ILD patients on nintedanib being followed up in outpatient clinics of the Rheumatology Department of the Fourth Department of Internal Medicine, Hippokration General Hospital of Thessaloniki, Greece, from June 2019 to November 2022. All patients were receiving nintedanib for CTD-ILD according to the recommendation of the treating physician. Due to the anonymized and non-interventional nature of the study, ethics approval was not required. CTD-ILD was identified using high-resolution computed tomographic scans, reduced values of FVC (% of the predicted value) and diffusion capacity of the lungs for DLco (% of the predicted value), and via clinical manifestations of worsening dyspnea. Nintedanib was started on patients with progressive fibrosing ILD as previously defined [17,30,32]. PFTs in routine examination and in clinical trials mainly focus on FVC, DLco, and forced expiratory volume in 1 s (FEV1) measurements [33,34,35]. The % of the predicted values of the FVC, Dlco, and FEV1 was preferred as it is considered a primary outcome value and provides an adjusted, reliable, and simple prognostic factor [36,37]. Patients with coexisting obstructive pulmonary disease or previous treatment with other antifibrotic agents such as pirfenidone were not included in the analysis. All patients were receiving nintedanib in the approved doses of 150 mg bd or 100 mg bd and the therapeutic value of the treatment was evaluated by lung function tests requested by the treating physician.

### 2.2. Data

Data that was collected from the outpatient clinic’s record included demographic information (sex, age), smoking status (all patients were nonsmokers), duration of CTD-ILD, type of autoimmune disease, serological profile, and presence of other systemic manifestations. The FVC (% of the predicted value), the DLco (% of the predicted value) and FEV1 (% of the predicted value) before and after treatment with nintedanib, the FVC% difference and DLco% difference after treatment, and the percentage change of the % predicted value of FVC and Dlco for every patient were also recorded. The initial measurements of FVC and FEV1 were in L, and the initial measurements of DLco were in mL/min/mmHg. Additionally, we recorded FVC%/DLco% ratio measurements before and after nintedanib treatment, as a result >1.5 might be a predictor of pulmonary arterial hypertension [38]. Other data that was documented includes the follow-up month (defined as the month that the patient individually repeated pulmonary function tests after the treatment initiation and was different for every patient); the C-reactive protein (CRP) level and erythrocyte sedimentation rate (ESR) before and after the treatment; adverse effects; and concomitant treatment with steroids and disease-modifying drugs.

### 2.3. Statistical Analysis

Data were handled anonymously. Normally distributed variables are presented as mean and standard deviation (sd). Non-normally distributed variables are presented as the median and interquartile range (IQR). The normality of the variables’ distribution was checked with the Shapiro–Wilk test. Differences in baseline variables were tested with t-tests for continuous variables. The homogeneity of variances was tested via Levene’s test. Wilcoxon’s test was used for categorical variables that were normally distributed and the Mann–Whitney U test was used for categorical variables that were not normally distributed. The comparisons between two continuous variables were conducted via univariate linear regression models. The effect of one or more (continuous or categorical) independent variables on the values of a continuous dependent variable was also examined via linear regression models. The individual variables from the univariate analyses that met the criterion of *p*-value < 0.2 were also analyzed in multivariate models. The level of statistical significance was set at *p*-value < 0.05 unless otherwise noted. The statistical analysis was performed using the SPSS statistical package version v27.

## 3. Results

### 3.1. Population

A total of 21 CTD-ILD patients were included in the study. Of these, 14 were female, all were nonsmokers, and the median age was 64 years (IQR = 9, marginal values: min = 29 years, max = 77 years). The mean duration of CTD-ILD was 4.8 years (sd = 2.5 years, marginal values: min = 1 year, max = 10 years). The CTDs of the patients were by order of prevalence: systemic sclerosis (SSc) 43% (*n* = 9), rheumatoid arthritis (RA) 23% (*n* = 5), dermatomyositis (DM) 19% (*n* = 4), juvenile rheumatoid Arthritis (JRA) 5% (*n* = 1), undifferentiated connective tissue disease (UCTD) 5% (*n* = 1), and interstitial pneumonia with autoimmune features (IPAF) 5% (*n* = 1). The marker autoantibodies of the patients were by order of prevalence: Scl-70 42% (*n* = 9), rheumatoid factor (RF) 19% (*n* = 4), Jo1+ 19% (*n* = 4), ro+ 10% (*n* = 2), anti-CCP 5% (*n* = 1), and anti-ENA screening 5% (*n* = 1). The majority of patients, 57% (*n* = 12), also had other systemic manifestations, which were: 14% (*n* = 3) gastrointestinal disorders such as gastroesophageal reflux disease, 14% (*n* = 3) cardiac disorders such as arrythmias, 14% (*n* = 3) pulmonary arterial hypertension, 1% (*n* = 5) digital ulcers, 1% (*n* = 5) skin disease, and 1% (*n* = 5) SICCA symptoms (xerostomia, xeropthalmia). Eighty-six percent (*n* = 18) of the patients were receiving other concomitant treatments besides nintedanib due to the underlying connective tissue disease which were: prednisolone 67% (*n* = 14), mycophenolate mofetil 48% (*n* = 10), calcium channel blockers 19% (*n* = 4), endothelin receptor inhibitors 19% (*n* = 4), methotrexate 14% (*n* = 3), tocilizumab 14% (*n* = 3), hydroxychloroqine 10% (*n* = 2), sildenafil 10% (*n* = 2), abatacept 5% (*n* = 1), azathioprine 5% (*n* = 1), and rituximab 5% (*n* = 1). The clinical and serological characteristics of the participants as well as concomitant medications are presented in Table 1 and Table 2.

### 3.2. Effect of Nintedanib in Pulmonary Function Tests

**Figure 1:**
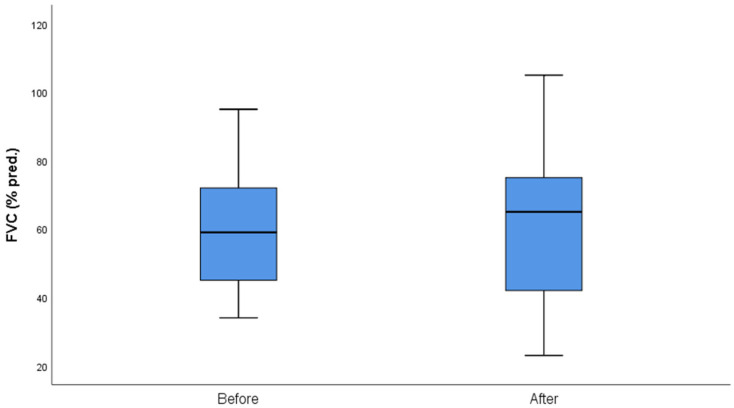
FVC (% pred) before and after nintedanib treatment.

**Figure 2:**
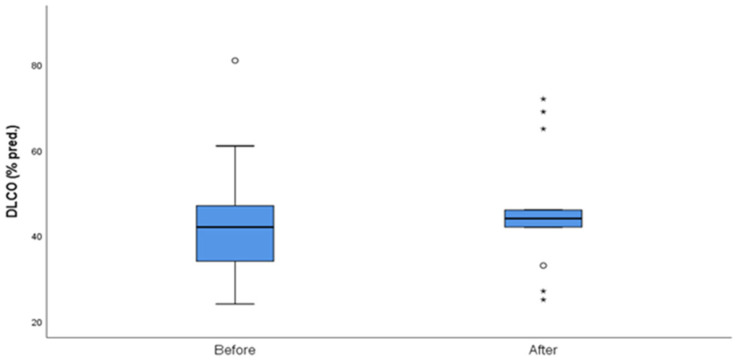
DLCO (% pred) before and after nintedanib treatment. Outliers are marked with a circle and extreme outliers are marked with a star.

**Figure 3:**
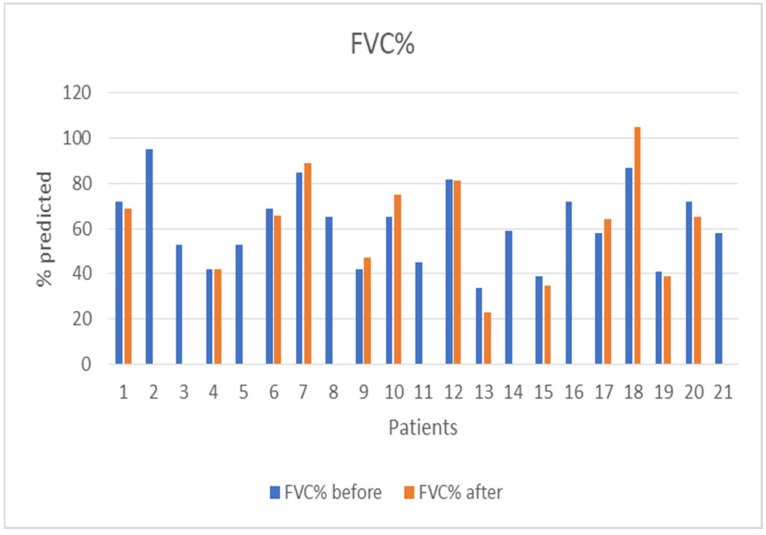
Patients’ FVC (% pred) before and after nintedanib treatment.

**Figure 4:**
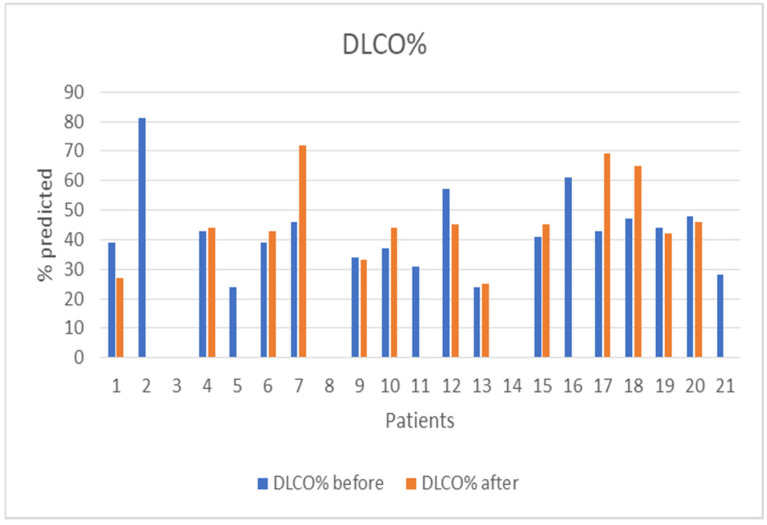
Patients’ DLco (% pred) before and after nintedanib treatment.

The median follow-up of the patients after initiation of nintedanib was 10 months (IQR = 5, min = 6 months, max = 27 months). During the follow-up period, two (10%) patients discontinued treatment due to gastrointestinal (GI) side effects, predominantly severe nausea and stomachache, two (10%) patients died due to severe CTD, three (14%) patients discontinued for unknown reasons, and one (5%) has not been followed-up yet for unknown reasons. Some patients included in the analysis could not perform a before-treatment DLco measurement due to COVID-19 restrictions, although the spirometry examination was conducted as usual. As expected, no statistical difference was demonstrated in FVC%, DLco%. FEV1% and FVC%/DLco% values before and after nintedanib treatment for the remaining 13 patients included in the analysis. However, the results indicate the stability and deceleration of deterioration of PFTs. In particular, PFTs are consistent, the mean FVC% difference is mildly increased by +0.9 (sd = 7.6), whereas the mean DLco% difference is increased by +3.4 (sd = 12.6) and the mean FEV1% presented a numerical improvement of +3.4%. The percentage change of FVC% is −0.3% (sd = 13.9) and the percentage change of DLco (% Pred) is +7.6% (sd = 27.1), suggesting stabilization of pulmonary function (Table 3). The mean FVC%/DLco% ratio presented a reduction of −0.1, suggesting a reduction in the score that indicates the risk of developing pulmonary arterial hypertension. The PFTs’ values before and after treatment for the whole group are presented in Figure 1 and Figure 2, while Figure 3 and Figure 4 present the relevant data for each patient.

### 3.3. Inflammatory Markers

Regarding patients’ inflammatory markers, no statistical difference was demonstrated in the CRP and ESR values before and after nintedanib treatment, which is as expected because nintedanib is not considered as an anti-inflammatory regimen. The median CRP and ESR after treatment presented numerical improvements of 0.1 mg/dL and 16.5 mm/h, correspondingly. The inflammatory markers’ values before and after treatment are presented in Table 3.

### 3.4. Side Effects

Regarding adverse effects, two (10%) patients developed serious, intolerable nausea and discontinued nintedanib treatment, and one (5%) patient developed diarrhea that was managed with dietary changes and antidiarrheal drugs.

### 3.5. Additional Analysis

**Figure 5:**
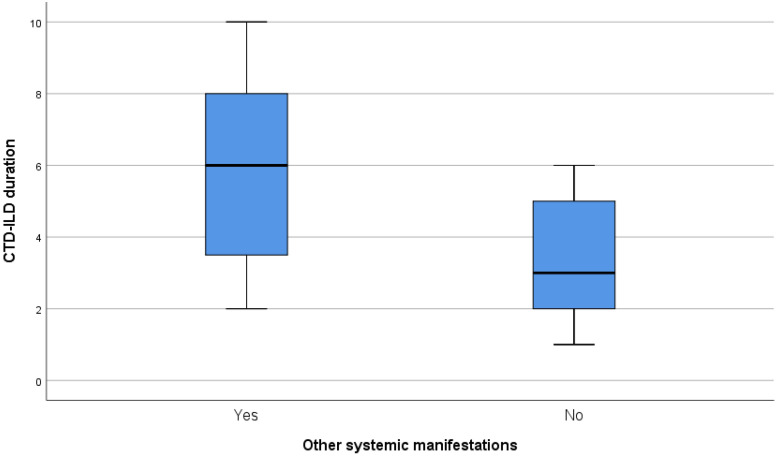
Relationship of CTD-ILD duration and other systemic manifestations.

Supplemental exploratory objectives included correlations between FVC% (before and after treatment), DLco% (before and after treatment), FVC%/DLco% difference, CTD-ILD duration, and the recorded data via statistical tests or univariate and multivariate linear regression when the criteria were met. Statistical significance was found between CTD-ILD duration and other systemic manifestations (*p*-value = 0.015). As expected, a significantly important increase in the mean duration of CTD-ILD of 2.5 years was observed in patients with extrapulmonary involvement, highlighting the cumulative effect of inflammation on various organs in patients with CTD. The relationship between CTD-ILD duration and other systemic manifestations is presented in Figure 5. Moreover, we conducted two subgroups analyses for the most frequent concomitant medications our patients received, prednisolone and MMF. A *t*-test was conducted in order to evaluate the FVC% difference (before and after nintedanib treatment) and DLco% difference (before and after nintedanib treatment) between patients that received prednisolone or not and between patients that received MMF or not. A significant mild improvement in DLco% measurements was noticed in the group that received MMF (*p*-value = 0.03), enhancing the importance of the coadministration of nintedanib with immunosuppressive drugs. Additionally, we added univariate linear regressions for all the concomitant treatments and multivariate linear regressions for the concomitant treatments that met the criterion of *p*-value < 0.2. The results of the univariate analyses confirmed the result of the subgroup analysis regarding MMF. The results of the multivariate analysis were not significantly important.

## 4. Discussion

CTD-ILD has been recognized as an emerging organ involvement with a high burden in terms of mortality and associated morbidity, with an average lifespan of almost less than five years [39], not only amongst individuals with SSc but also across the whole spectrum of systemic diseases, especially rheumatoid arthritis and dermatomyositis. Subsequently the introduction of novel licensed therapies targeting fibrotic processes is of major interest to both patients and physicians dealing with this population. Despite the severity of this clinical entity, there are still limited data concerning CTD-ILD and nintedanib [40]. In this regard, the findings of our study confirm the positive effect of nintedanib in halting the progression rate of pulmonary fibrosis in patients with chronic fibrosing CTD-ILD, suggesting that antifibrotic treatment could complement immunosuppressives in the management of lung fibrosis.

The results of our cohort concur with previous findings in randomized, double-blind, placebo-controlled trials. In the INBUILD trial, in which 25% of patients were diagnosed with CTD-ILD and progressive fibrosing phenotype, treatment with nintedanib culminated in a lower rate of FVC decline compared to placebo [17] which was consistent across the subgroups of patients with systemic autoimmune disorders regardless of the underlying disease, namely RA, SSc, or mixed CTD [41]. The SENSCIS trial investigated the efficacy of nintedanib in SSc-ILD and reported a lower rate of FVC decline as well [30]. In contrast, real-world data relating to the efficacy of nintedanib in individuals with CTD-ILDs are limited to a small report on three cases [42] whilst the majority of published evidence refers to other types of ILDs, predominantly idiopathic and familiar [43,44]. In this regard, the findings of the current study further expand previous observations by confirming the beneficial effects of nintedanib in a larger group of CTD-ILD patients.

The main CTDs represented in our population were RA and SSc, similar to the distribution of participants in the INBUILD trial [41] as both diseases convey a high prevalence of ILD manifestations and severe lung fibrosis [45]. Moreover, in patients with SSc, particular autoantibody patterns and systemic sclerosis subtypes (diffuse or limited) have been linked to a more severe organ involvement and mortality rate. Scl-70-positive patients and patients with diffuse systemic sclerosis have a higher chance of developing clinically severe ILD. Subgroup analyses from the SENSCIS trial concluded that nintedanib treatment is efficient at stabilizing pulmonary deterioration across all SSc subgroups, independently of the Scl-70 positivity status and SSc categorization [46]. However, about 20% of our patients were diagnosed with Jo-1 positive dermatomyositis, which represents an emerging entity associated with aggressive and refractory to treatment ILD [47]. In this subgroup the administration of nintedanib was also effective and safe, in line with a larger real-life retrospective study of inflammatory-myopathy-ILD which also showed that nintedanib may improve survival in this population [48].

More than half of the patients in our study were suffering from multiorgan involvement due to the underlying CTD, such as gastrointestinal dysfunction, cardiac disorders, and pulmonary arterial hypertension. The link between severe ILD and several comorbidities, mostly related to the cardiac and gastrointestinal systems, has been previously described [49], particularly in SSc patients in whom gastroesophageal reflux disease is strongly associated with the pathogenesis and the severity of pulmonary fibrosis [50]. In this respect, the correlation between ILD duration and other organ involvement signifies the severity of lung fibrosis in our population and further supports the favorable effects of nintedanib in slowing the progression of fibrosing ILD in high-risk patients.

Given that glucocorticoids and immunomodulatory medications are the hallmark treatments for systemic autoimmune disorders, the majority of our patients (86%) were also under immunosuppressive treatment. As expected, the most common concomitant medications were corticosteroids (prednisolone < 7.5 mg/day) and MMF. Fewer patients were treated with biologic drugs such as tocilizumab, abatacept, and rituximab, as indicated by recent studies [51,52,53,54]. Data from the SENSCIS trial strongly suggest that the co-administration of immunosuppressive drugs and nintedanib may provide the greatest efficacy in slowing FVC decline in SSc-ILD patients. The characteristics of our patients are similar to those treated with nintedanib and immunosuppressive drugs in randomized controlled trials, indicating that the two treatments may complement each other, confirming the correlation of concomitant therapy with MMF in stabilizing PFTs [55]. The outcomes of our study confirm the efficacy and safety of combination therapy across a broad range of conventional and biologic disease-modifying drugs commonly and empirically administrated in patients with CTD-ILD in daily clinical practice.

The adverse effect profile in our study was characterized by gastrointestinal manifestations, mainly severe and resistant nausea, which led two older patients to discontinue treatment. In contrast, diarrhea presented in about 5% of the patients and was manageable with practical recommendations (diet modification, antidiarrheal use), enabling patients to maintain the use of nintedanib. The two deaths recorded in our population were attributed to critical CTD, and the remaining missing follow-up was irrelevant to side effects. In summary, the safety profile of nintedanib in our cohort was consistent across different subgroups of CTD-ILD and with what has been reported in randomized trials [56]. Of note, discontinuation due to severe nausea is more common in elderly patients, as occurred in our study [57,58]. The two deaths during the study were due to myocardial involvement in an SSc patient, and to ILD exacerbation in a RA patient who could not tolerate nintedanib.

This study has some limitations. Since it is an observational and descriptive study, it cannot draw many conclusions on causality and effect. Moreover, the small patient group may lead to temporal and unsafe associations. There was no control group as it was not ethical to include a placebo parallel group once a therapy has been approved as the standard of care. Additionally, the evolution of symptoms and imaging progression were not considered in this study. Furthermore, there were no fixed follow-up appointments or standardization of the follow-up process, as it was regulated by the individual patient’s visit schedule. Many patients delayed their follow-up due to restrictions associated with the COVID-19 pandemic, and the lockdown disruption may have led to missing or delayed data. However, this study includes CTD-ILD patients across the whole spectrum of autoimmune disorders in concomitant treatment with other immunosuppressives in a real-life setting.

## 5. Conclusions

ILD remains a severe and difficult-to-treat complication of systemic autoimmune disorders with an unfavorable impact on patients’ quality of life and prognosis. The results of the current study provide real-world evidence of the beneficial effect of nintedanib in slowing the rate of fibrosis progression across the whole spectrum of patients with CTD-ILD that were parallelly treated with other anti-inflammatory and immunosuppressive drugs. The adverse effect profile consisted mostly of gastrointestinal manifestations, especially nausea in elderly patients with a low drop-out rate related to this symptom, consistent with relevant studies. Further targeted randomized controlled trials are needed to assess the efficacy of nintedanib in CTD-ILD.

## Figures and Tables

**Table 1:** Patients’ characteristics.

Parameter	Values
N	21
Age (years), Median (IQR)	64 [min = 29, max = 77] (9)
Male, *n* (%)	7 (33%)
SSc, *n* (%)	9 (43%)
RA, *n* (%)	5 (23%)
DM, *n* (%)	4 (19%)
JRA, *n* (%)	1 (5%)
UCTD, *n* (%)	1 (5%)
IPAF, *n* (%)	1 (5%)
CTD-ILD-duration (years), mean (sd)	4.8 [min = 1, max = 10] (2.5)
Other systemic manifestations-yes, *n* (%)	12 (57%)
GI disorders, *n* (%)	3 (14%)
Cardiac disorders, *n* (%)	3 (14%)
PAH, *n* (%)	3 (14%)
Ulcers, *n* (%)	1 (5%)
Skin disease, *n* (%)	1 (5%)
SICCA (xerostomia, xeropthalmia), *n* (%)	1 (5%)
Scl-70 +, *n* (%)	9 (42%)
RF +, *n* (%)	4 (19%)
jo1 +, *n* (%)	4 (19%)
Ro +, *n* (%)	2 (10%)
Anti-CCP +, *n* (%)	1 (5%)
Anti-ENA +, *n* (%)	1 (5%)
Follow-up visit (in months), Median (IQR)	10 (5)
Concomitant Treatment-yes, *n* (%)	18 (86%)

IQR interquartile range, SSc systemic sclerosis, RA rheumatoid arthritis, DM dermatomyositis, JRA juvenile rheumatoid arthritis, UCTD undifferentiated connective tissue disease, IPAF interstitial pneumonia with autoimmune features, CTD-ILD connective tissue interstitial lung disease, GI gastrointestinal, PAH pulmonary arterial hypertension.

**Table 2:** Types of concomitant treatment.

Categories	Treatment	Values
Immunosuppressives	Prednisolone, *n* (%)	14 (67%)
	Mycophenolate mofetil, *n* (%)	10 (48%)
	Methotrexate, *n* (%)	3 (14%)
	Tocilizumab, *n* (%)	3 (14%)
	Hydroxychloroquine, *n* (%)	2 (10%)
	Abatacept, *n* (%)	1 (5%)
	Azathioprine, *n* (%)	1 (5%)
	Rituximab, *n* (%)	1 (5%)
Vasodilators	Calcium channel blockers, *n* (%)	4 (19%)
	Endothelin receptor inhibitors, *n* (%)	4 (19%)
	Sildenafil, *n* (%)	2 (10%)
Other regimens	Proton pump inhibitors, *n* (%)	15 (71%)

**Table 3:** Patients’ diagnostic tests before and after nintedanib treatment.

Diagnostic Test	Value	*p*-Value
FVC-BEFORE (% Pred), Mean (sd)	61.3 (17.4)	0.67
FVC-AFTER (% Pred), Mean (sd)	61.5 (23.4)	
FVC-DIFFERENCE (% Pred), Mean (sd)	0.9 (7.6)	-
Percentage change of FVC (% Pred), Mean (sd)	−0.3 (13.9)	-
DLco-BEFORE (% Pred), Mean (sd)	42.6 (13.8)	0.21
DLco-AFTER (% Pred), Mean (sd)	46.2 (14.6)	
DLco-DIFFERENCE (% Pred), Mean (sd)	3.4 (12.6)	-
Percentage change of DLco (% Pred), Mean (sd)	+7.6 (27.1)	-
FEV1 BEFORE (% Pred), Mean (sd)	66.2 (16.06)	0.89
FEV1 AFTER (% Pred), Mean (sd)	69.6 (19.9)	
FVC%/DLco% BEFORE, Mean (sd)	1.5 (0.39)	0.45
FVC%/DLco% AFTER, Mean (sd)	1.4 (0.47)	
CRP BEFORE (mg/dl), Median (IQR)	0.7 (0.8)	0.26
CRP AFTER (mg/dl), Median (IQR)	0.6 (0.92)	
ESR BEFORE (mm), Median (IQR)	25 (17)	0.44
ESR AFTER (mm), Median (IQR)	16.5 (43.5)	

FVC forced vital capacity, DLco diffusion capacity of the lungs for carbon monoxide, FEV1 forced expiratory volume in 1 s, CRP C-reactive protein, ESR erythrocyte sedimentation rate.

## Data Availability

Not applicable.
